# TSSAR: TSS annotation regime for dRNA-seq data

**DOI:** 10.1186/1471-2105-15-89

**Published:** 2014-03-27

**Authors:** Fabian Amman, Michael T Wolfinger, Ronny Lorenz, Ivo L Hofacker, Peter F Stadler, Sven Findeiß

**Affiliations:** 1 Bioinformatics Group, Department of Computer Science and the Interdisciplinary Center for Bioinformatic, University of Leipzig, Härtelstraße 16–18, 04107 Leipzig, Germany; 2 Institute for Theoretical Chemistry, University of Vienna, Währingerstraße 17, 1090 Vienna, Austria; 3 Center for Integrative Bioinformatics Vienna (CIBIV), Max F. Perutz Laboratories, University of Vienna, Medical University of Vienna, Dr. Bohr-Gasse 9, 1030 Vienna, Austria; 4 Department of Biochemistry and Molecular Cell Biology, Max F. Perutz Laboratories, University of Vienna, Dr. Bohr-Gasse 9, 1030 Vienna, Austria; 5 Center for RNA in Technology and Health, University of Copenhagen, Grønnegårdsvej 3, Frederiksberg C, Denmark; 6 Max Planck Institute for Mathematics in the Sciences, Inselstraße 22, D-04103 Leipzig, Germany; 7 Fraunhofer Institute for Cell Therapy and Immunology, Perlickstraße 1, D-04103 Leipzig, Germany; 8 Santa Fe Institute, 1399 Hyde Park Road, Santa Fe NM 87501, USA; 9 Research group Bioinformatics and Computational Biology, Faculty of Computer Science, University of Vienna, Währingerstraße 29, 1090 Vienna, Austria

**Keywords:** Differential RNA sequencing, dRNA-seq, TSS, Transcription start site annotation, Transcriptome, RESTful Web service, Next generation sequencing

## Abstract

**Background:**

Differential RNA sequencing (dRNA-seq) is a high-throughput screening technique designed to examine the architecture of bacterial operons in general and the precise position of transcription start sites (TSS) in particular. Hitherto, dRNA-seq data were analyzed by visualizing the sequencing reads mapped to the reference genome and manually annotating reliable positions. This is very labor intensive and, due to the subjectivity, biased.

**Results:**

Here, we present TSSAR, a tool for automated *de novo* TSS annotation from dRNA-seq data that respects the statistics of dRNA-seq libraries. TSSAR uses the premise that the number of sequencing reads starting at a certain genomic position within a transcriptional active region follows a Poisson distribution with a parameter that depends on the local strength of expression. The differences of two dRNA-seq library counts thus follow a Skellam distribution. This provides a statistical basis to identify significantly enriched primary transcripts.

We assessed the performance by analyzing a publicly available dRNA-seq data set using TSSAR and two simple approaches that utilize user-defined score cutoffs. We evaluated the power of reproducing the manual TSS annotation. Furthermore, the same data set was used to reproduce 74 experimentally validated TSS in *H. pylori* from reliable techniques such as RACE or primer extension. Both analyses showed that TSSAR outperforms the static cutoff-dependent approaches.

**Conclusions:**

Having an automated and efficient tool for analyzing dRNA-seq data facilitates the use of the dRNA-seq technique and promotes its application to more sophisticated analysis. For instance, monitoring the plasticity and dynamics of the transcriptomal architecture triggered by different stimuli and growth conditions becomes possible.

The main asset of a novel tool for dRNA-seq analysis that reaches out to a broad user community is usability. As such, we provide TSSAR both as intuitive RESTful Web service ( http://rna.tbi.univie.ac.at/TSSAR) together with a set of post-processing and analysis tools, as well as a stand-alone version for use in high-throughput dRNA-seq data analysis pipelines.

## Background

Deep sequencing approaches were successfully applied to examine the architecture of primary bacterial transcriptomes and uncovered an unexpectedly complex architecture [1-5]. Although plain transcriptome sequencing can in principle be sufficient to determine transcription start sites (TSS) as local accumulations of read starts, this approach requires extensive sequencing depth [6,7]. Alternative TSS located within well-expressed genes or operons remain undetectable since moderate changes in coverage do not offer a sufficiently distinctive signal. On the other hand, TSS are not the only loci at which read starts accumulate in RNA-seq data. Alternative sources of such signals are specific processing sites, secondary structures that influence RNA degradation patterns, or chemical modifications [8-10].

The differential RNA sequencing method dRNA-seq [4] is designed to overcome these difficulties. It makes use of the 5’-monophosphate dependent terminator RNA exonuclease (TEX) that specifically degrades processed RNA, which exhibits a monophosphate at its 5’ end. Transcription initiation, in contrast, produces a 5’-triphosphate that protects the unprocessed 5’ end from degradation by TEX. Treating RNA isolates with TEX prior to reverse transcription to cDNA, leads to a sequencing library ([+]-library or treated library) that is enriched in primary transcription starts, compared to an untreated total RNA library ([–]-library or untreated library). Similar to other library preparation steps that enrich or deplete certain transcript types, e.g. TAP treatment [11] and rRNA depletion [12], the TEX dependent degradation of processed RNA fragments is not perfect. The [+]-library, therefore, still contains a mixture of primary and processed transcripts, albeit with a distribution of read starts that is shifted significantly towards TSS positions [4]. In the data used in this contribution a median enrichment at TSS positions of 3.5 is observable. The discrimination of TSS from other accumulations of read starts is thus non-trivial and cannot be performed unambiguously from a TEX treated library alone. On the other hand comparison of [+]- and [–]-libraries offers a potentially highly informative source of information: while read starts will be relatively enriched, we can expect the alternative types of read start accumulations to be depleted in the [+]-library.

Since the signal at hand is quantitative rather than an all-or-none qualitative difference, it is imperative to employ a statistical model to assess when an observed enrichment is indeed significant. This depends strongly on the expression level. To distinguish between real TSS signals and accidental read start accumulation resulting from imperfect TEX degradation or high local expression, the aid of a background model, e.g. the [–]-library, is needed.

Hitherto, the analysis of the dRNA-seq data consists of mapping sequencing reads for each library onto the reference genome, visualizing the read coverage in a genome browser, often with displayed gene and transcription unit annotation, promoter predictions and other available prior knowledge. With this background the genome is manually inspected for positions with a more pronounced peak in the [+]- compared to the [–]-library. The interpretation of dRNA-seq signals in such a way is not only very time consuming, tedious, and error-prone, but also highly subjective and weakly reproducible. Additional annotation information from third-party sources can be very helpful but bear the risk to introduce biases, resulting in re-annotation of already "known" features, and neglecting signals that are less obviously associated with current annotation data. It is, therefore, preferable to separate dRNA-seq data analysis from subsequent data integration with additional available information.

To overcome these shortcomings we developed TSSAR (*TSS* *A*nnotation *R*egime), a tool for automated *de novo* TSS annotation from dRNA-seq data. Incorporation of information like gene annotation or promoter predictions is deferred to post-processing steps.

## Implementation

### Theory

Detailed knowledge of the underlying background distribution is required to quantify the significance of differential read start count signals. Although related, this problem differs from the thoroughly examined problem of describing the variance in read counts per gene, which is routinely applied in the process of differential gene expression analysis. On one hand, the background is variable along the genome, depending on the transcription activity of the considered region. On the other hand, the distribution of read starts within an equally transcribed region depends on many concomitants. These are met by the different steps in the RNA-seq library construction, namely cDNA production by reverse transcriptase, fragmentation (enzymatic or mechanic), adapter ligation, read amplification by PCR, size selection, and finally the chemistry of the sequencing platform itself. Since the technology and the protocol details vary and develop with a compelling rate, it is far from trivial to capture these details [13]. Therefore, it is sensible to recollect the basic characteristic of RNA-seq data, which basically constitute count data. With this simplification we can assume that the distribution of read starts within an expressed genomic region can be modeled by a Poisson distribution with parameter *λ*. Given *λ* the Poisson probability $P(Y=k)=\frac{\lambda^{k}e^{-\lambda }}{k!}$ describes the probability that *k* reads start at a genomic position. In dRNA-seq data genomic positions with significantly enriched differences between the Poisson distributions of [+]- and [–]-library are potential TSS. Therefore, we are concerned with finding positions where the observed difference cannot be explained easily by the local model of the background expression in the [–]-library. The difference of two Poisson distributions is given by the Skellam distribution [14] with the cumulative distribution function

(1) $$\begin{array}{l} F(D,\lambda_{\text{[+]}},\lambda_{\text{[–]}})=\overset{D}{\underset{d=-\infty }{\sum }}e^{-(\lambda_{\text{[+]}}+\lambda_{\text{[–]}})}{\left(\frac{\lambda_{\text{[+]}}}{\lambda_{\text{[–]}}}\right)}^{\frac{k}{2}}I_{\left|k\right|}(2\sqrt{\lambda_{\text{[+]}}\lambda_{\text{[–]}}}) \end{array}$$

Here *λ*_[+]_ and *λ*_[–]_ are the parameters describing the average read start rate in the [+]- and the [–]-library, respectively. *I*_|*k*|_ is the modified Bessel function of the first kind and integer order |*k*| [15].

A major practical issue is the estimation of the parameters *λ*_[±]_ for the two libraries. We assume that read start counts per position within transcriptional active regions follow a Poisson distribution, with the expected value *λ* depending on the transcription rate, or to be more precise, on the RNA abundance, which depends on the transcription rate and the RNA stability. Within untranscribed regions the read start count per position is ideally zero. In reality, this idealization is often not met due to "leaky" promoters, read-through from adjacent genes, spurious transcription starts at random positions, sample contamination, and sequencing errors leading to mis-mapping of individual reads. Nevertheless, as a first approximation it is reasonable to neglect these sources of error since the effect can be regarded to be small compared to the coverage dispersion within transcribed regions.

As a consequence, randomly selected genomic regions, which are most likely a mixture of untranscribed and transcribed regions, can be modeled with a mixture model of a Poisson distribution and a background that is 0 with probability 1. To separate the two underlying distributions and estimate the parameter *λ*, describing only the transcriptionally active region, a zero-inflated Poisson model regression [16,17] is applied. For each sample *Y* the probability *ϕ* that an observed zero is an excess *structural zero*, is estimated, such that

(2) $$P(Y=0)=\phi +(1-\phi )\cdot e^{-\lambda }$$

where *e*^-*λ*^ is the probability for a position within the Poisson distributed part to have zero reads starting there (*sampling zero*). These positions are part of transcriptional active regions. We use a zero-inflated Poisson regression to estimate *ϕ* and thus determine how many positions without read starts are structural and sampling zeros, respectively. Only the latter and positions that have at least one read start are used to estimate *λ* of the [+]- and [–]-library, respectively. The estimation of *λ* thus effectively considers the transcriptionally active regions only. In practise, the removal of structural zeros leads to larger estimates for *λ* and thereby avoids the incorrect prediction of TSS from small counts in regions with low numbers of observed read starts.

### Program architecture

TSSAR has been implemented in Perl and R and is available in two variants: A stand-alone version incorporates the core statistic routines and is best suited to be used in custom high-throughput dRNA-seq analysis. The Web service (available at http://rna.tbi.univie.ac.at/TSSAR/) comprises additional components for pre- and post-processing, thus providing a Web-based, cross-platform compatible pipeline for dRNA-seq analysis. An overview of the pipeline workflow can be found in Additional file 1: Figure S1.

**Figure 1:**
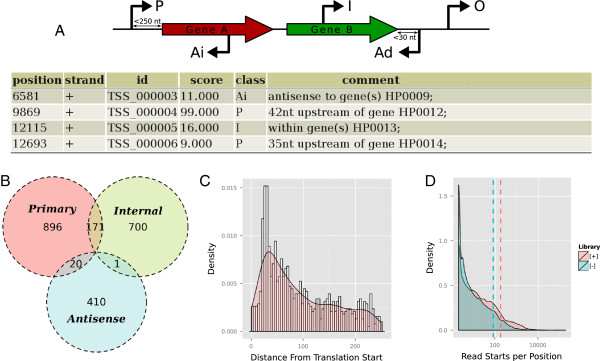
**Post-processing and Visualization.** **(A)** Similar, but more restrictive, to the scheme in [4] each annotated transcription start site is classified according to its genomic context: If a TSS is positioned within 250 nt upstream of an annotated gene, it is classified as **P**rimary. TSS within an annotated gene is labeled **I**nternal. A TSS which is on the opposite strand of an annotated gene is classified as **A**ntisense. This class further splits into **Ai** and **Ad**, for internal antisense and downstream antisense, respectively. The latter is reserved for a TSS which points in the opposite reading direction and is less than 30 nt downstream of an annotated gene. A TSS that falls in none of these classes is reported to be **O**rphan. **(B)** As a matter of fact, one TSS can have several labels as it might fall into more than one of the aforementioned classes. The TSSAR Web service summarizes the counts of the overlapping main classes graphically. **(C)** For TSS which are annotated as ’Primary’ the 5’UTR lengths are deduced and the corresponding distribution is plotted. **(D)** To assess the efficiency of the TEX treatment, the distribution of read starts per position is provided as a helpful indicator. If the enrichment in the [+]-library worked efficiently, we expect fewer read start sites, each of which will have more reads. Hence the distribution is flattened on the left side and bulged at the right side. The corresponding distribution and the mean (dashed line) is expected to be shifted to the right compared to the [–]-library.

The **TSSAR Web service** is built on top of the Perl Dancer[18] framework and adheres to the Representational State Transfer (REST) [19] principles of Web architecture. The first step in using the TSSAR online pipeline is pre-processing of mapped reads, i.e., extracting the essential information of read start counts per genomic position. To avoid the necessity of uploading huge mapping files (typically for bacterial genomes up to several gigabytes), we implemented the **TSSAR client** for local pre-processing of mapped reads in SAM/BAM or BED format on the user’s computer. To grant platform independence, the TSSAR client is implemented in Java. Once the relevant data is extracted from the mapping files assisted by the Picard tools[20], files are compressed using XZ utils[21] and automatically transferred, using the Apache HttpComponents[22] package, to the TSSAR Web server. On the Web server the statistical calculations are conducted and potential TSS are predicted. The TSSAR Web service provides an assortment of post-processing steps. The list of predicted TSS can be reduced by merging consecutive TSS and cluster them into the most prominent position. For samples where the reference genome annotation was specified, all annotated TSS are classified into primary, internal, anti-sense or orphan, according to their position relative to nearby genes, see Figure 1A. Based on the classification the 5’ UTR length distribution is determined. All results are visualized and provided for download. Figure 1 depicts partly the output for showcase data sets [4,23]. Beside the shown results, the output additionally contains all annotated TSS and the clustered TSS list in BED [24] and GFF format. All tables are available in comma and tab-separated lists, as excel and HTML files. With the assistance of the pre-computed plots, it is easy to gain a quick overview of the quality of the analysis.

While the TSSAR Web service provides convenient usability for routine dRNA-seq analysis tasks, there is also a demand for integrating third-party bioinformatics tools into custom analysis pipelines. To address this issue, we provide a **TSSAR stand-alone version**. In this version, the implementation is restricted to processing of SAM files, analysis based on the statistical calculations, and output of annotated TSS in BED format. The stand-alone version is available for download from the TSSAR Web site.

### Statistical calculation

We chose a sliding window approach with a dynamic assessment of each position in the context of its local surrounding in order to account for different transcription rates across the genome. As a matter of fact, the choice of the window size parameter has an effect on the results (see Additional file 1: Figure S2). There, two conflicting interests have to be balanced. On the one hand, the region should be large enough to provide enough information for a reliable distribution parameter estimation. On the other hand, the region should be small enough to provide an as homogeneous surrounding as possible. If the sliding window covers more than one actively transcribed gene, with different RNA abundances, the variance will be estimated over all transcribed entities. This might blur small signals, e.g., for low abundant sRNA genes. As a compromise, the default window size is 1,000 nt, approximately matching the average length of prokaryotic genes. It can be easily adjusted by the user.

For each window the parameters describing the Poisson distribution are estimated in the following manner: First, the sample values are winsorized [25], i.e., the highest read start count is substituted with the second highest count. The same procedure is done for the lowest value. This increases the robustness of the method against outliers, which may be caused by mis-mapping and/or abundant RNA fragments e.g. arising from rRNA loci.

**Figure 2:**
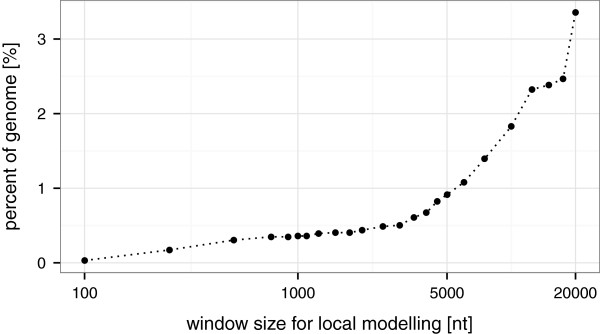
**Regions of non-convergence.** Regions where the applied zero-inflated Poisson regression does not converge are omitted from the analysis and need manual inspection. Since the basic unit which cannot converge is the step size (equals a tenth part of the windows size) there is a correlation between the parameter window size and the percentage of the genome which can not be modeled. The *H. pylori* dRNA-seq data (see section Evaluation) shows that for all practical useful window sizes below 5,000 nt, less then 1% of the genome eludes analysis.

Second, the zero-inflated Poisson regression is applied to estimate *ϕ*, the probability that an observed zero is a *structural zero* from an untranscribed region instead of a *sampling zero* from a transcribed region. The R package VGAM is used for the regression [17,26]. Here, the parameters describing the Poisson distribution are fitted by full maximum likelihood estimation (MLE). In case the MLE algorithm fails to converge, which might happen because the underlying assumption of a well behaved Poisson distribution is violated, the respective window is excluded from further analysis. While this might seem to be a drawback, it serves as a minimal plausibility check, ensuring the data fulfills the underlying assumption of following a Poisson distribution. Sequencing libraries with low complexity but many PCR duplicates might otherwise feign confidence in the results, which can actually not be deduced from the data. A BED file listing the omitted segments which typically correspond to less than 1% of the genome is provided (see Figure 2). In the evaluation data set (see section Evaluation) modeled with a window size of 1,000, 24 regions with a total length of 12,000 bases could not be modeled (∼0.5% of the genome). The majority correspond to tRNA and rRNA coding loci (10 and 5 single regions, respectively). Additionally, 4 regions overlapped with annotated protein coding genes and the remaining 5 did not overlap with any annotated gene. A manual screening of the corresponding regions revealed that they share common characteristics. Generally, they are small islands with very high expression levels.

Third, a regression procedure is applied to each window in the [+]- and in the [–]-library separately. For each library the probability *ϕ* is transformed into an expected number of excess structural zeros. Since the same genomic region is under consideration in both libraries, a similar proportion of untranscribed and transcribed regions can be expected. To increase robustness, the average between the number of structural zeros in both libraries is calculated and the estimated number of zeros are removed from each library. To determine *λ* for each library, describing the Poisson distribution of the sample, the arithmetic mean of the remaining counts is calculated.

In the next step the probability that the read start differences between [+]- and [–]-library can be explained by the aforementioned background model is calculated. For this purpose, the original read start counts are normalized by

(3) $$\begin{array}{l} {\hat{p}}_{i}=\left\{\begin{array}{ll} p_{i}\cdot \frac{\sum M}{\sum P} & \text{if}\sum M>\sum P\\ p_{i}\cdot 1 & \text{if}\sum M\leq \sum P \end{array}\right) \end{array}$$

(4) $$\begin{array}{l} {\hat{m}}_{i}=\left\{\begin{array}{ll} m_{i}\cdot \frac{\sum P}{\sum M} & \text{if}\sum P>\sum M\\ m_{i}\cdot 1 & \text{if}\sum P\leq \sum M \end{array}\right) \end{array}$$

Thereby, *p*_*i*_ and ${\hat{p}}_{i}$ are the raw and normalized values of the [+]-library at position *i*, respectively. The same applies to the [–]-library, i.e., *m*_*i*_ and ${\hat{m}}_{i}$. ∑P and ∑M are the native sums of all read start counts in the total [+]- and [–]-library, respectively. The effect of this step is to scale the read counts of the larger library relative to the smaller one, hence avoiding artificial distending of the sample variance. The estimated parameters *λ*_[+]_ and *λ*_[–]_ are therefore normalized accordingly.

For each sequence position *i* in the current window, the difference ${\hat{d}}_{i}={\hat{p}}_{i}-{\hat{m}}_{i}$ of the normalized counts between [+]- and [–]-library is calculated. Unexpectedly large positive values of ${\hat{d}}_{i}$ for position *i* indicate TSS, while exceptional negative values may indicate processing sites. The probability of observing ${\hat{d}}_{i}$ is evaluated w.r.t. the Skellam distribution with the estimated normalized Poisson parameters.

The window slides along the genome with a step size equal to 1/10^*th*^ of the window size, hence each position is evaluated in 10 slightly different contexts. The geometric mean of all ten *p*-values is calculated in order to obtain the final position-wise *p*-value. Finally, each position that falls below a user-specified average *p*-value cutoff and whose total read start count in the [+]-library exceeds a user specified noise cutoff is reported as a significant TSS. The noise cutoff serves as an additional safeguard to restrict the results to plausible annotations. This is needed because the Skellam distribution works only with the differences of the expectation values of the underlying Poisson distributions. A very high expectation value in the [–]-library in combination with a small expectation value in the [+]-library leads to a negative expectation value of the resulting Skellam distribution. This, in turn could lead to annotated positions which are not supported by reads in the [+]-library, as significantly enriched. To prevent this unwanted behavior a user defined number of read starts must be observed in the [+]-library.

## Results

The goal of the TSSAR method is to provide user-friendly tools for rapid annotation of significant TSS based on dRNA-seq data. We therefore implemented a stand-alone version and a Web service. The first is intended to be used in high-throughput analysis pipelines whereas the latter represents an easy to use and platform independent user interface. For a Web service it is important to avoid the transfer and storage of gigabyte-sized mapping files. We therefore provide a Java client that extracts the necessary information and asks the user for only two parameters, namely genome size and window size. The data is pre-processed locally on the user’s computer. The essential information, i.e., the number of sequencing reads starting at each position, is automatically uploaded and analyzed on the TSSAR Web server (see Additional file 1: Figure S1). All relevant cutoffs like *p*-value and noise threshold are subsequently selectable for precomputed values.

### Evaluation

To evaluate the performance of TSSAR in analyzing dRNA-seq data, we resort to the published data set for *Helicobacter pylori*[4]. We used the publicly available raw sequencing data from the Sequence Read Archive [27] (study accession number SRP001481), restricting ourselves to the dRNA-seq data from mid-logarithmic growth phase and acid stress growth condition. The reads were pooled and mapped to the reference genome (NCBI accession ID NC_000915) using segemehl version 0.1.4 [28] with default parameters.

Based on this data, which were normalized in the same way as indicated in equations 3 and 4, we predicted putative TSS with three different approaches. The first two represent a naïve benchmark. First, we calculated the difference ( ${\hat{p}}_{i}-{\hat{m}}_{i}$) for each position *i* of the [+]- and [–]-library read start counts. We applied different cutoff thresholds between 1 and 300, thereby denoting every position with a difference higher than the cutoff to be a putative TSS. The resulting list of potential TSS was compared to the manual annotation from [4] using BEDTools Intersect[29], allowing ±2 nt inaccuracy to call a manual and an automated annotated TSS the same. The second approach is quotient based. Analogous to the difference based approach, the quotient $\frac{{\hat{p}}_{i}+1}{{\hat{m}}_{i}+1}$ is calculated for each position *i* (+1 is used as pseudo-count to avoid division by zero). Again we use different cutoff values between 1.1 and 20. These two approaches have their static nature in common. The same threshold is applied for the whole genome. A similar approach was already applied by [30]. Albeit, there it was used to identify differentially induced TSS between different strains and growth conditions and additional information about promoter sequences was used to gain specificity.

Finally, we applied the dynamic TSSAR model, which analyzes the transcriptome locally and thus is able to model the different dynamics within the transcriptome. Here, we used a window size of 1,000 nt (approximately the mean gene length in *H. pylori*) and a noise cutoff of 3 reads per position. We filtered with different *p*-value threshold from 1·10^-15^ to 9·10^-1^.

**Figure 3:**
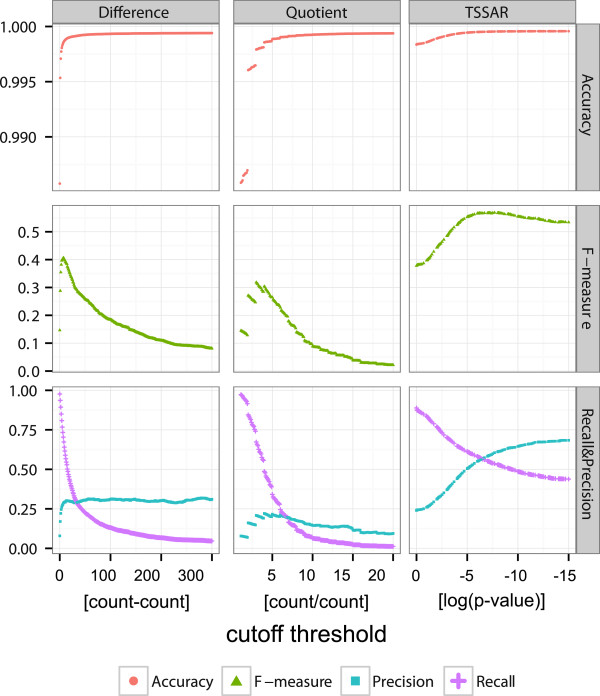
**Evaluation of** TSSAR**performance.** Comparison of the prediction power of TSSAR against two fixed-cutoff approaches *Difference* and *Quotient*. For each method different cutoff thresholds were applied. The difference, quotient and logarithm of the *p*-value are plotted along the *x*-axis. Please note, for comparability the log(*p*-value) is plotted in descending order from left to right. The resulting predictions were evaluated by calculating the recall rate, precision, F-measure and accuracy. The dynamic approach of TSSAR clearly outperforms the remaining in all aspects. Since only TSSAR applies a clustering of consecutive TSS positions, this effect was separately examined, results can be found in Additional file 1: Figure S5.

From these results, each threshold based prediction is evaluated using standard measurements: recall rate ( $\frac{\text{TP}}{\text{TP}+\text{FN}}$), precision ( $\frac{\text{TP}}{\text{TP}+\text{FP}}$), accuracy ( $\frac{\text{TP}+\text{TN}}{\text{TP}+\text{FP}+\text{FN}+\text{TN}}$) and the F-measure ( $2\times \frac{\text{precision}\times \text{recall}}{\text{precision}+\text{recall}}$) [31], where *TP*, *TN*, *FP* and *FN* are true positive, true negative, false positive and false negative predictions, respectively. Figure 3 depicts the results of this analysis. TSSAR shows a much higher precision and simultaneously a less sharp decrease of the recall rate. In terms of the F-measure, it outperforms the fixed-threshold approaches by about 2-fold. A further major advantage is the smoother course of the F-measure along different *p*-value cutoffs. This makes the resulting annotation less dependent on the cutoff choice. The optimal cutoff value for the basic annotation strategies based on difference or ratio might be very variable for different experiments and difficult to deduce without a reference annotation.

In its default settings TSSAR merges consecutive TSS. Since the tested naïve approaches do not share this behavior, we tested the influence of TSS clustering on the prediction performance separately (see Additional file 1: Figure S5). Although, clustering contributes to the precision of the prediction, the effect is too small to cause the improved performance of TSSAR.

**Figure 4:**
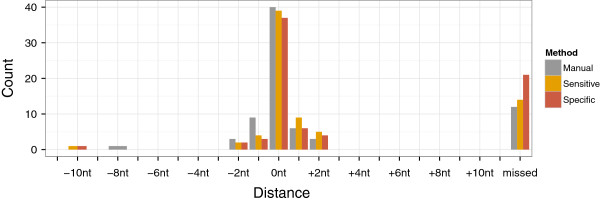
**Recall experimental validated TSS.** Comparison of 74 experimentally validated TSS described in literature [4] with TSSAR results. The *Manual* TSS annotation recovered 40, 15 and 6 TSS with a 0, ±1 and ±2 nt offset, respectively. Here 12 TSS were annotated more than 10 nt away from the experimentally determined position (summarized as *missed* in the plot). TSSAR was run with a *Sensitive* and a *Specific* parameter set (*p*-value cutoff 0.05 and 0.0001; noise cutoff 1 and 3, respectively). With sensitive parameters 39 TSS (53%) were annotated on the exact same position. Of the remaining TSS 13 and 7 were annotated with ±1 and ±2 nt variance, respectively, whereas 14 TSS (19%) were annotated more than 10 nt away. The specific TSSAR prediction annotated 37, 9 and 6 TSS with 0, ±1 and ±2 nt offset, respectively, relative to the experimentally validated position. In this case 21 TSS (28%) were annotated more than 10 nt away, and therefore annotated as missed. The results of the same analysis including also our naïve benchmark approaches can be found in Additional file 1: Figure S3.

Additionally, besides comparing our automated annotation to the manual annotation by the authors, we examined how precise TSSAR reproduces known *H. pylori* TSS. Therefore, we used TSS studied in detail by independent methods, such as primer extension or 5’ RACE. From the 74 examples described in the literature (summarized in Additional file 1 of [4]), we calculated the distance to the closest position which we annotated as TSS. If the discrepancy was more then 10 nt, we considered the TSS as not recovered. Figure 4 shows the result of this analysis for two TSSAR annotations with different parameters. The first one with lenient threshold values (aiming for sensitivity), and the later with more stringent values (aiming for specificity). In both cases the majority of experimentally confirmed TSS could be detected at the exact same position (39 and 37 TSS, respectively). TSSAR missed 14 and 21 TSS, respectively, compared to the 12 TSS that were also not detectable in the manual annotation by the authors of [4]. We have to emphasis that, in contrast to a manual annotation, our method is not aware of any annotation information, which might induce a human curator to prefer certain positions. Comparison of the two naïve approaches and TSSAR emphasizes that the presented statistical method is relatively insensitive to certain parameter thresholds, see Additional file 1: Figure S3.

During manuscript preparation a new method for dRNA-seq data analysis, called TSSpredator, became available. It combines a peak calling approach for the treated library and a ratio based approach between the treated and untreated library [32]. Since the parameters for this method were trained on the very same data set we used for the evaluation, the performance of methods cannot be impartially compared on this data. We can report, however, that TSSAR’s statistical method performs equally well even on this set (Additional file 1: Figure S6). To demonstrate that TSSAR has more generally applicable default parameters, we compared the two methods on a new, publicly available dRNA-seq data set from *Stenotrophomonas maltophilia*[33]. Based on the mapped reads (treated and untreated dRNA-seq data from the WT strain, SRS352126 and SRS352125, mapped with segemehl read aligner [28] against the reference genome NC_010943, considering only uniquely mapped reads), TSSAR and TSSpredator were applied each in the default settings.

**Figure 5:**
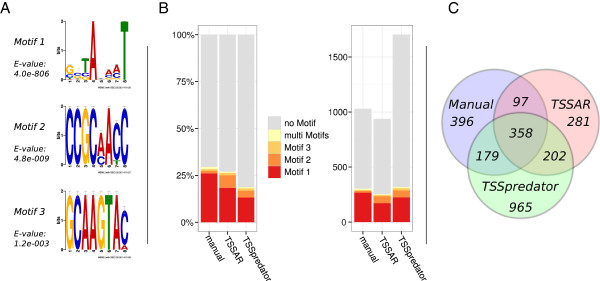
**Comparison** TSSAR**and** TSSpredator**.** To assess the performance of TSSAR and TSSpredator we used dRNA-seq data of *S. maltophilia*[33]. Thereby, the enrichment of cis-regulatory DNA motifs upstream of the predicted TSS was used as a surrogate for sensitivity. Furthermore, the individual results were compared to a manual annotation. Panel **A** shows the significantly enriched sequence motifs. Panel **B** shows the relative enrichment and the total count of this motifs in the sets of all TSS predicted by TSSAR, TSSpredator and by a manual analysis. Panel **C** depicts the overlap of the TSS annotated by the different methods.

The TSS predictions, together with the authors’ manual annotation taken from the supplementary data of [33] were analysed for congruency (Figure 5C), and for enrichment of conserved sequence motifs that may be constitute promoter elements (Figure 5A,B). To this end, we extraced the 20 nt upstream regions of the putative TSS reported by TSSAR (938 TSS), TSSpredator (1704), and manual annotation (1030). The combined set was then screened for overrepresented sequence motifs with MEME[34]. We found three motifs with *E* < 0.001. Their position weight matrices (PWMs) are shown in Figure 5A. The Pribnow box [35] like Motif 1 is very similar to the dominant promoter motif determined for *Xanthomonas campestris* in [5], a close relative of *S. maltophilia*.

The PWMs of these motifs were then mapped back to the putative promoter regions with FIMO[36]. We counted a motif found if the *p*-value of the hit was below 0.001, Figure 5B. For TSS annotated by TSSAR 27.1% are associated with one of the three motifs, of which the majority corresponds to the Pribnow box like motif, while only 18.7% of the TSSpredator results are associated with a motif. The manualy curated list of TSS shows a motif for 29.5% of the entries.

The reproduction of the manual annotation on the *S. maltophilia* data shows an F-measure of 0.46 and 0.39 for TSSAR and TSSpredator, respectively. Which is mainly due to the fact that the latter predicts about 1,100 additional TSS whereas TSSAR only predicts about 500 not manually annotated TSS, Figure 5C.

These results indicate a higher accuracy of the TSSAR analysis. TSSAR reproduces manual annotation more precise and TSS annotated by TSSAR exhibit a higher enrichment in putative promoter motifs, indicating its greater specificity compared to TSSpredator. However, the latter results have to be interpreted with caution since several recent studies, e.g. [37,38], showed that the textbook knowledge of the homogeneous architecture of promoter region motifs does not capture the full complexity of biological reality.

## Discussion

A major advantage of an automated TSS annotation, based on a sound statistical analysis, neglecting *a priori* knowledge of the whereabouts of promoters and other already established annotation, lies in the avoidance of any bias towards certain genomic positions. This ensures an unbiased analysis as well as a comparable and reproducible TSS annotation procedure.

Although our approach checks whether the basic assumption that the read starts of a sequencing library are Poisson distributed holds, a manual inspection of the produced data is still recommended. The automated TSS prediction is only as good as the underlying dRNA-seq libraries. We therefore emphasize that a thoughtful investigation of the input sequencing reads, especially for PCR duplicates, is advised. Manual inspection is necessary for those genomic regions that are not annotated by TSSAR due to non-convergence in the estimation of the expression parameters. For TSSAR’s output, we recommend at least a basic sanity check, since very complex regions, such as tRNA and rRNA loci, might be misconstrued. In spite of these precautions, the work load to check hundreds or a few thousands of predicted TSS positions is significantly reduced compared to screening millions of genomic positions in the first place.

Reliable and automated TSS annotation is a prerequisite for many applications. So far, most genome-wide TSS annotations focused on a static picture of the transcriptomal architecture [2,39] (there are also notable exceptions, e.g. [30,40]). One reason is that data analysis was more laborious than data generation. Relieving the experimenter from this time-consuming burden might liberate the resources to investigate more of the dynamics and alteration of the transcriptome, due to external stimuli or evolutionary differences. During manuscript preparation the latter was demonstrated by conducting a comparative transcriptomics approach [32]. There, TSS annotation was also conducted in an automated manner. First, putative TSS are selected by considering the "flank height", and the differences of mapped read starts of position *i*-1 to *i* are calculated. These sites are then evaluated similarly to our *Quotient* approach based on the ratio between the TEX treated and untreated library. The problem of selecting an educated cutoff, which is immanent to all methods but especially troublesome for classifiers which directly depend on variable conditions such as sequencing depth, was neatly circumvented by using a comparative approach. Transcriptomes of different *Campylobacter jejuni* isolates were used to dynamically adjust thresholds if signals in different strains could be observed. In the more typical application scenarios, where such comparative information is not available, a robust *p*-value estimate that takes the dynamic range of transcription activity along the whole genome into account for the classification seems to be preferable.

Currently, TSSAR is based on the assumption that a [+]- and [–]-library is analyzed and only positions with a significant enrichment in the [+]-library are reported as potential TSS. At least two other application scenarios of the statistical framework are possible. One is to detect RNA processing sites and the other to analyze differentially induced transcription starts. In principle the latter could be achieved by comparing two TEX treated libraries resulting from dRNA-seq runs of different growth conditions. In that case, a large positive and negative ${\hat{d}}_{i}$ is of importance as it indicates (growth phase dependent) induction of a TSS in the one or the other library. RNA processing sites are in principle detectable using the "standard" dRNA-seq approach. Positions where a significant enrichment in the [–]-minus over the [+]-library is observable are of interest. Extremely small values of ${\hat{d}}_{i}$ point to these positions. Tackling both issues, processing sites and induced transcription initiation, is however currently hampered by the lack of experimentally verified training sets. Furthermore, although tailored for analyzing dRNA-seq data, in principle, the TSSAR method should be applicable to other RNA-seq protocols, e.g., [11], which aim to enrich read starts at certain positions in the sequencing library. Currently, the run-time of TSSAR, see Additional file 1: Figure S4, prevents its application for one of the above mentioned purposes to complete eukaryotic genomes. An improvement of this aspect will be a task for the future development and refinement of the program.

The modularity of the TSSAR framework makes it possible to extend the current approach e.g., by improving the statistical model. Alternative approaches based on a different (non-Poisson) distribution or the Pitman sampling method [6] can be implemented in the TSSAR core module, without the necessity to change the Java client or the Web service front end. The RESTful architecture of the TSSAR Web service provides additional extensibility, rendering implementation of new functionality such as promoter or operon characterization straightforward.

## Conclusion

Here, we presented an automated analysis of dRNA-seq data which aims to detect significantly enriched TSS positions. The background distributions of sequencing read starts are modeled locally by a zero inflated Poisson distribution. Positions with a larger difference between the TEX treated and the untreated library than expected, considering the background, are annotated as significant transcription start sites. We could show that our method reproduces manually analyzed dRNA-seq data better than two simple approaches that use a global cutoff to discriminate between true and false signals. Furthermore, the choice of a *p*-value cutoff is more intuitive and less arbitrary.

TSSAR is available both as a stand alone tool and as a Web service at http://rna.tbi.univie.ac.at/TSSAR/. The latter provides additional post-processing functionality like TSS classification or merging of consecutive TSS. The TSSAR Web service offers user-friendly and intuitive online access to the TSSAR framework whereas the stand-alone version is intended for integration into third-party annotation pipelines.

## Availability and requirements

• **Project name:** TSSAR

• **Project home page:**http://rna.tbi.univie.ac.at/TSSAR

• **Operating system:** Platform independent

• **Programming language:** Java, Perl and R

• **Other requirements:** Client needs Java 1.6 or higher and the standalone version is based on Perl 5, R 2.15

• **License:** Java client under Apache License, Statistics module under GPL2.

• **Any restrictions to use by non-academics:** For non-profit use only.

## Competing interests

The authors declare that they have no competing interests.

## Authors’ contributions

FA implemented the statistical analysis and evaluated the performance, SF programmed the Java client, MTW, FA and RL implemented the Web service. FA, MTW, RL, ILH, PFS and SF contributed to the implementation details and testing, collaborated in writing and approved the final manuscript.

## Supplementary Material

Additional file 1 **Supplementary Information**. File that contains supplementary information, i.e. additional figures. Click here for file
