# Supplementary material for: TSSAR: TSS annotation regime for dRNA-seq data
Source: BMC Bioinformatics. 2014 Mar 27;15:89. doi: 10.1186/1471-2105-15-89 (PMC4098767; doi:10.1186/1471-2105-15-89)
Supplement: Additional file 1 — Supplementary Information. File that contains supplementary information, i.e. additional figures. [file 1471-2105-15-89-S1.pdf]

Supplementary information: "TSSAR: TSS Annotation Regime for dRNA-seq data"

Fabian Amman, Michael T. Wolfinger, Ronny Lorenz, Ivo L. Hofacker,
Peter F. Stadler and Sven Findeiß

February 26, 2014

1 Supplementary Figures

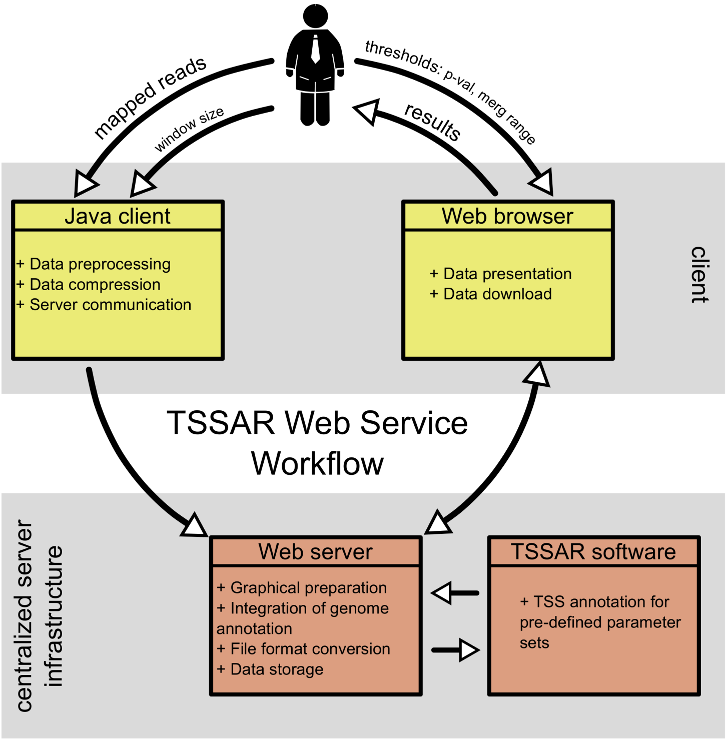

Supplementary Figure 1: **Web service architecture.** The user interacts on one hand with the Java client, providing mapped dRNA-seq reads in bam/sam format. On the other hand, after the computational analysis the results can be screened in the Web browser. Here, the user can interact by adjusting different parameters, i.e., the p-value cutoff, the merge range to cluster consecutive TSS, and the noise cutoff. In the background, the Web server orchestrates different programs to analyze and graphically represent the data. Among others, the core program `TSSAR` is applied to make the statistical calculation of the enrichment p-values for each genomic position.

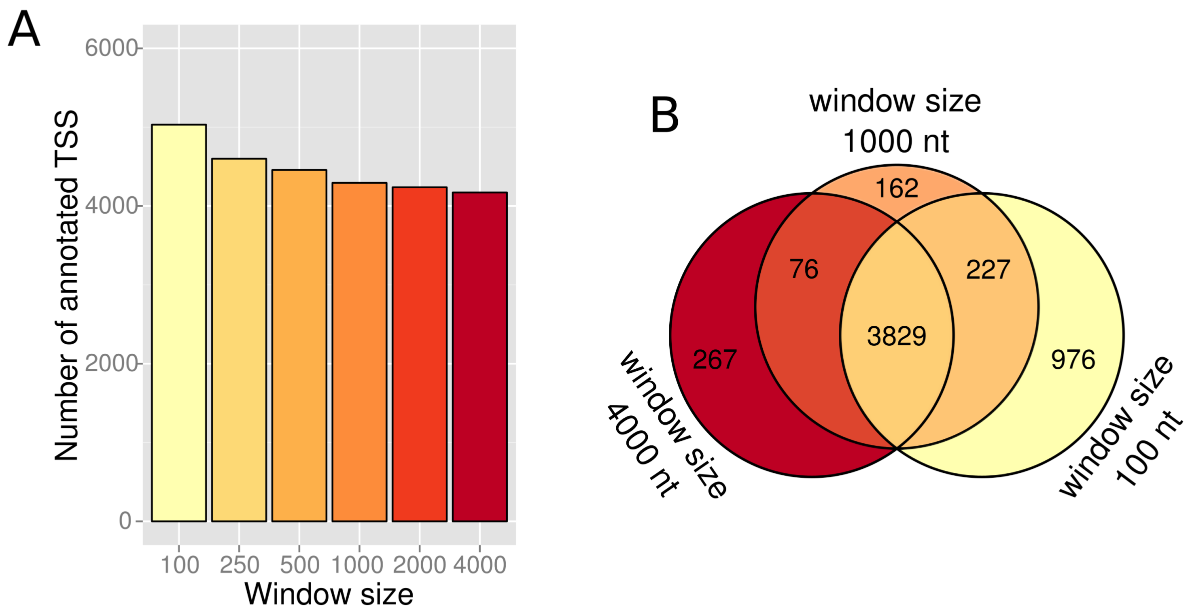

Supplementary Figure 2: **Effect of window size.** `TSSAR` tests each position in the context of its local surrounding. Thereby, the extent of this region is determined by the window size parameter. (**A**) depicts the relation between window size and the number of annotated TSS for a dRNA-seq sample from *B. pertussis*. The smaller the window size the more positions are annotated as TSS. (**B**) shows the overlap between the different predictions for the three examined window sizes. The majority of TSS is found by all three methods. An extremely small window size, such as 100 nt, tends to annotate significantly more TSS. This can be explained by the fact that a too small window provides too little information for estimating the underlying distribution parameter precisely enough. On the other hand, choosing a too large window size results in the parameter estimation over a heterogeneous region, leading to parameters which are too small for highly expressed and too big for lowly expressed sub-regions, e.g. different transcripts. Therefore, as a compromise the average gene length in the considered species is suggested as the most reliable window size.

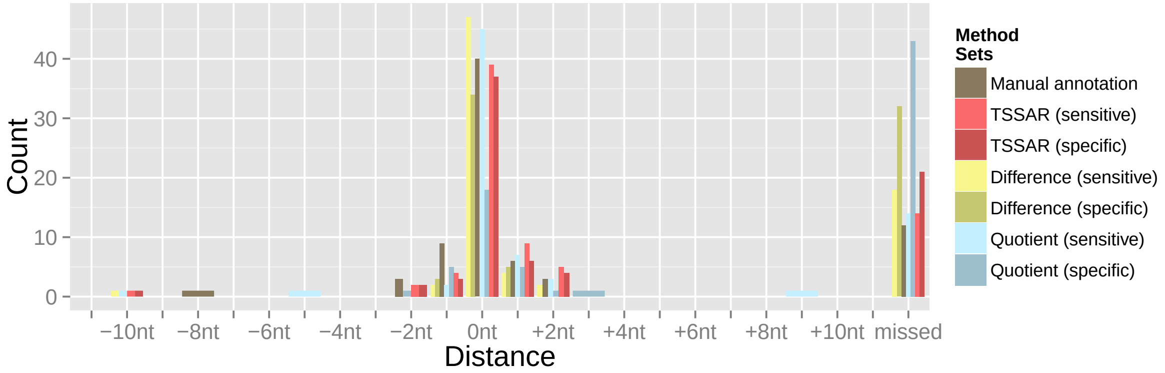

Supplementary Figure 3: **Recall experimental validated TSS.** Comparison of 74 experimentally validated TSS described in literature. In contrast to Figure 4, here the recall rate of the two naïve approaches *Difference* and *Quotient* are also included. The thresholds were +3 and +30 for sensitive and specific mode of the difference based approach and 2-fold and 4-fold for the quotient based approach, respectively. The plot shows, in accordance with Figure 3 the high recall rate for very sensitive threshold choices, and the performance decline for higher thresholds. This points to the difficulty of reasonable threshold choices for static approaches and the more robust behavior of the presented dynamic, thus context sensitive, statistical method implemented in `TSSAR`.

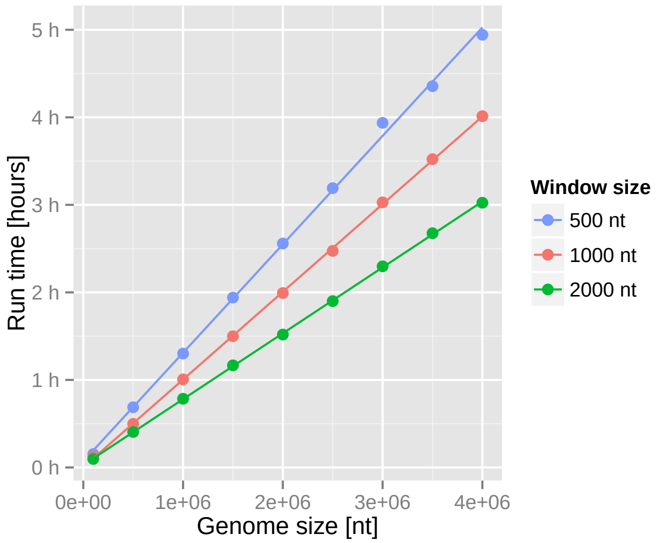

Supplementary Figure 4: **CPU time requirements.** The run-time is almost exclusively dependent on the genome size and the window size, since the algorithm applies the sliding window from the start to the end (=genome size), even if no reads map to a particular region. We tested the time needed to process different subsets of a dRNA-seq data set from *B. pertussis* with a genome size of >4 Mbp. Therefore, a standard desktop workstation (Intel® Core™ i5-3570K CPU @ 3.40GHz; 32GB RAM) was used with the `TSSAR` stand alone version. As a rule of thumb, the time requirement to process 1 Mb of genomic sequence at the default window size of 1000 nt is approximately one hour.

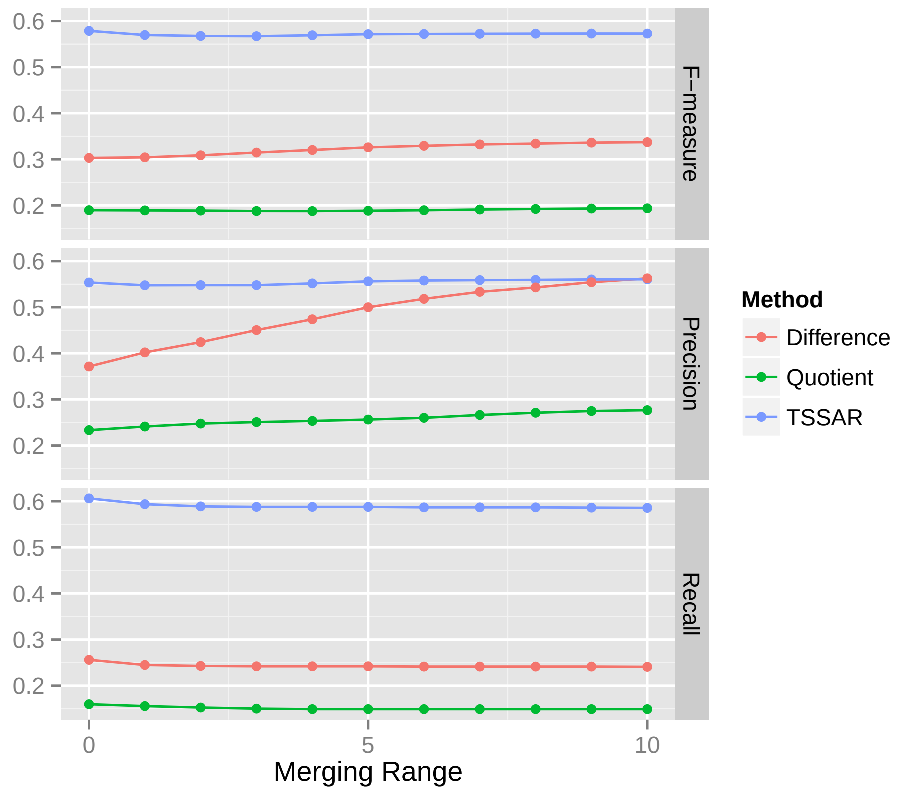

Supplementary Figure 5: **Effect of clustering onto performance.** For the methods used in Figure 3 to evaluate the performance, the effect of merging consecutive TSS up to a maximal distance, was evaluated. Therefore, each method was used to analyze the same data set (*H. pylori*). Applied thresholds are, a difference of ≥ 20, a quotient of ≥ 4 and a p-value $\leq 10^{-6}$, respectively. As expected, the merging has no effect on the recall rate. The precision is improving as the merge range is expanded, because the total number of annotated TSS is reduced without a change in the recall rate. `TSSAR` shows the best performance F-measure wise, emphasizing the conclusion that `TSSAR` gains its distinguished performance not from the merging of consecutive TSS positions alone.

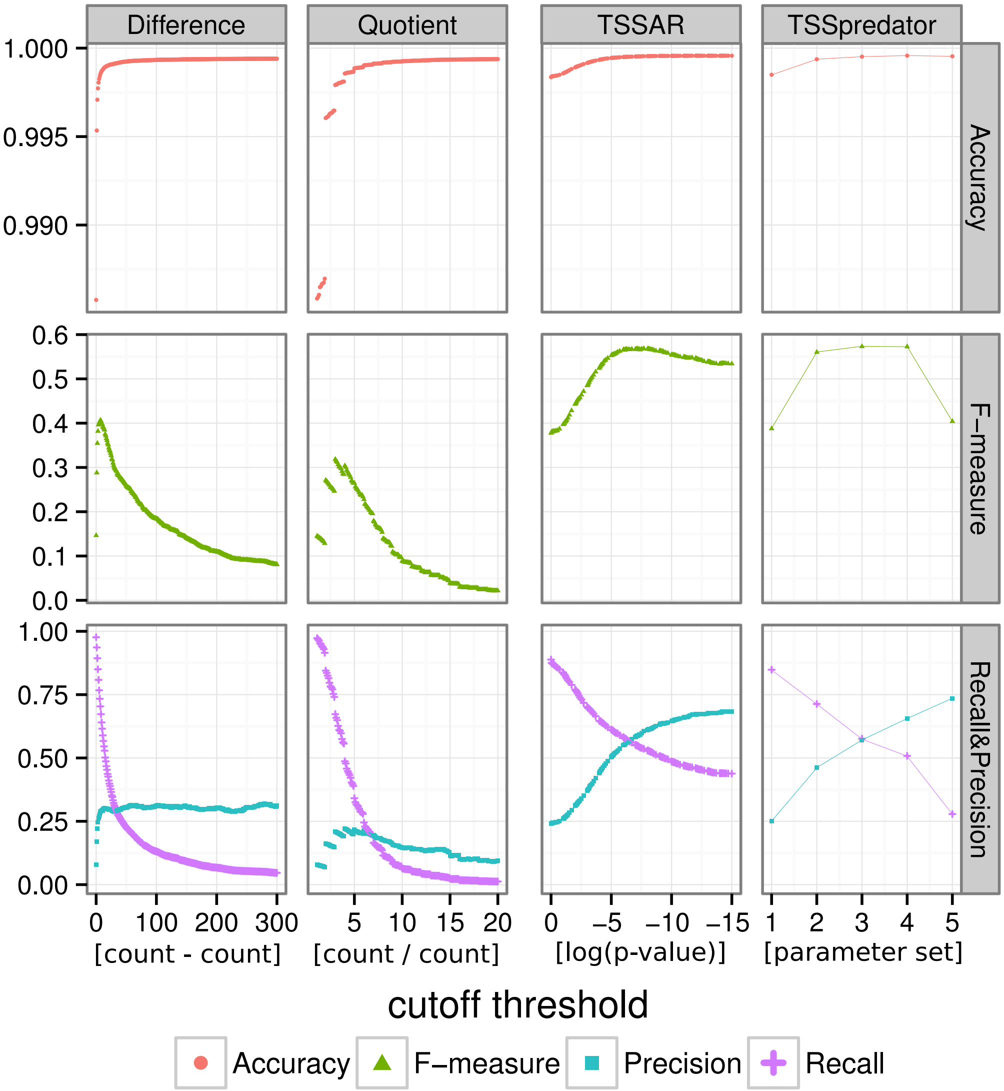

Supplementary Figure 6: **Evaluation of `TSSAR` performance including `TSSpredator`.** The same plot as depicted in Figure 3 extend by `TSSpredator`. There, the five pre-defined parameter sets (1. . . *very sensitive*; 2. . . *sensitive*; 3. . . *default*; 4. . . *specific*; 5. . . *very specific*) were used. Please note that the `TSSpredator` parameter sets were optimized specifically for the very same data set reused here for this analysis. In contrast, `TSSAR` was not optimized and still performs equally well for this data set.
